# Tobacco

**Published:** 1875-06

**Authors:** 


Tobacco.—Tobacco belongs to the class of narcotic and exciting substances, and has no food value. Stimulation means abstracted, not added, force. It involves the narcotic paralysis of a portion of the functions, the activity of which is essential to healthy life. It will be said that tobacco soothes and cheers the weary toiler, and solaces the overworked brain. Such may be its momentary effects, but the sequel can not be ignored. All such expedients are fallacious. When a certain amount of brain-work or hand-work has been performed, nature must have space to recuperate, and all devices for escaping from this necessity will fail. It is a bad policy to set the house on fire to warm our hands by the blaze. Let it, then, be clearly understood that the temporary excitement produced by tobacco is gained by the destruction of vital force, and that it contains absolutely nothing which can be of use to the tissues of the body. Tobacco adds no potential strength to the human frame. It may spur a weary brain or feeble arm to undue exertion for a short time, but its work is destructive, not constructive. It can not add one molecule to the plasm out of which our bodies are daily built up. On the contrary, it exerts upon it a most deleterious influence. It does not supply, but diminishes, vital force. It has been denied that tobacco leads to organic disease, but the evidence is very strong the other way, and it would be very remarkable if continued functional disturbance did not ultimately lead to chronic derangement of the organs; that it causes functional disturbance, no one dreams of denying; indeed, it has been remarked that no habitual smoker can be said to have a day's perfect health.

